# How does informal employment affect health and health equity? Emerging gaps in research from a scoping review and modified e-Delphi survey

**DOI:** 10.1186/s12939-022-01684-7

**Published:** 2022-06-21

**Authors:** Juyeon Lee, Erica Di Ruggiero

**Affiliations:** Dalla Lana School of Public Health, Division of Social and Behavioural Health Sciences, University of Toronto, 155 College Street, Room 408, M5T 3M7 Toronto, ON Canada

**Keywords:** Informal employment, Health inequities, Sustainable development goals

## Abstract

**Introduction:**

This article reports on the results from a scoping review and a modified e-Delphi survey with experts which aimed to synthesize existing knowledge and identify research gaps on the health and health equity implications of informal employment in both low- and middle-income countries (LMICs) and high-income countries (HICs).

**Methods:**

The scoping review included peer-reviewed articles published online between January 2015 and December 2019 in English. Additionally, a modified e-Delphi survey with experts was conducted to validate our findings from the scoping review and receive feedback on additional research and policy gaps. We drew on micro- and macro-level frameworks on employment relations and health inequities developed by the Employment Conditions Knowledge Network to synthesize and analyze existing literature.

**Results:**

A total of 540 articles were screened, and 57 met the eligibility criteria for this scoping review study, including 36 on micro-level research, 19 on macro-level research, and 13 on policy intervention research. Most of the included studies were conducted in LMICs while the research interest in informal work and health has increased globally. Findings from existing literature on the health and health equity implications of informal employment are mixed: informal employment does not necessarily lead to poorer health outcomes than formal employment. Although all informal workers share some fundamental vulnerabilities, including harmful working conditions and limited access to health and social protections, the related health implications vary according to the sub-groups of workers (e.g., gender) and the country context (e.g., types of welfare state or labour market). In the modified e-Delphi survey, participants showed a high level of agreement on a lack of consensus on the definition of informal employment, the usefulness of the concept of informal employment, the need for more comparative policy research, qualitative health research, and research on the intersection between gender and informal employment.

**Conclusions:**

Our results clearly identify the need for more research to further understand the various mechanisms through which informal employment affects health in different countries and for different groups of informal workers.

**Supplementary Information:**

The online version contains supplementary material available at 10.1186/s12939-022-01684-7.

## Background

Informal employment remains a significant social and public health problem globally. Informal workers refer to all persons in employment who, “by law or in practice, are not subject to national labour legislation, income taxation, social protection or entitlement to certain employment benefits” [1]. Although the concepts of informal sector, informal employment, and informal economy were first adopted in 1950s to illustrate economic development within the ‘developing’ world [2], it has become more apparent that informal employment has significant relevance to high-income countries (HICs) in addition to low- and middle-income countries (LMICs). A standard employment relationship, which is full-time, permanent, and part of a subordinate relationship with one employer, became less dominant in HICs with the rise of alternative work arrangements including but not limited to dependent self-employment such as ‘gig’ work, a short-term contract arrangement mediated by online platforms [3]. With the rise of such non-standard employment relationships, more workers are likely to work informally due to the lack of access to employment-related health and social protection as well as to statutory regulations to protect workers from hazardous and unhealthy working conditions and to ensure adequate wages for workers and occupational safety and health measures.

The COVID-19 pandemic has led to a growing interest in the health and health equity implications of informal employment. The COVID-19 and its containment measures have had disproportionate health and socioeconomic impacts on informal workers [4]. The pre-existing challenges faced by informal workers, including low wages, job insecurity, poor working conditions, and lack of benefits and insurance coverage, were clearly exposed during the pandemic through their direct and indirect impacts on health and have also been exacerbated by the measures put in place to contain the spread of the virus [5]. Furthermore, the pandemic has particularly highlighted how informal employment intersects with gender, race/ethnicity, sexual identity, etc. [6]. For example, occupational groups involving frequent contact with people in the workplace, such as care and domestic workers, are predominantly comprised of women and migrant workers [7]. They have not only been exposed to health-harming working conditions, but also felt more overburdened with rising unpaid care and domestic demands due to the closure of schools and formal day-care services.

Calls for policy-relevant research on informal employment continue. These include: the International Labour Organization (ILO) Recommendation 204 on the transition from the informal to the formal economy, which was unanimously adopted in the tripartite International Labour Conference in 2015, the United Nations (UN) Sustainable Development Goal (SDG) indicator 8.3.1 under Goal 8 (Decent Work and Economic Growth) which calls for regular monitoring of the magnitude of informal employment in all countries (UN, 2015). Other relevant SDGs include: SDG Goal 3 (Healthy Lives) with Target 3.8 on achieving universal health coverage and Target 3.9 on reducing the number of deaths and illnesses from hazardous chemicals and others. These targets require labour market and welfare state policies that ensure access to health care and occupational safety and health for informal workers. Moreover, SDG Goal 5 (Gender Equality) urges all countries to “recognize and value unpaid care and domestic work through the provision of public services, infrastructure and social protection policies” (Target 5.4), reflecting the gendered nature of informal employment where women are disproportionately represented in the informal economy. These SDGs are deeply interconnected and, ideally, should be addressed in an integrated and intersectoral way.

While many studies in LMICs report informal workers as having poorer working conditions and poorer health than their counterparts in formal employment, this literature has not been systematically evaluated to draw out the implications for future research and policy. Moreover, empirical evidence in HICs has only started to be published [8]. Particularly noteworthy is the fact that precarious employment, despite its popularity as a social determinant of health in research and policy contexts of HICs, has been mostly studied in the formal sector; less is known about informal employment, its level of precariousness, and its implications on health, gender, and equity [9]. Employment precariousness, by definition, “makes it difficult to earn a decent income; interferes with their opportunities for decent working conditions; and/or puts their health and well-being at risk in material ways” [10]. Employment precariousness is not necessarily equivalent to informal employment; rather, it is a cross-cutting condition of employment encompassing multiple dimensions including job insecurity, low wage level, lack of social benefits, and less power to unionize [11]. Although informal wage earners are more likely to have higher levels of precariousness than workers in the formal sector, the level of employment precariousness can be heterogeneous within and between the formal and informal sectors. Given that studies on informal employment and health are being conducted in increasing numbers in both LMICs and HICs, an analysis of available evidence pertaining to the health and health equity implications of informal employment is needed.

This study outlines the findings of a scoping review and a modified e-Delphi survey with experts which sought to map out existing knowledge and identify research gaps on the health and health equity implications of informal employment in both HIC and LMIC contexts.

### Conceptual framework

To guide our scoping review, we drew on micro- and macro-level frameworks on employment relations and health inequities developed by the Employment Conditions Knowledge Network (EMCONET), one of the nine Knowledge Networks established under the auspices of the World Health Organization (WHO)’s Commission on the Social Determinants of Health [11]. The micro- and macro-level frameworks were used as a conceptual framework to inform the scoping review questions and as a tool to analyze and synthesize empirical studies and identify gaps in research (see Benach, Muntaner [11] for a detailed explanation of the frameworks). The micro- and macro-level frameworks were originally developed for the purposes of helping us better understand the complex links between employment relations and health, guiding further observations and testing of potential mechanisms linking employment relations and health inequities, and helping identify potential policy interventions for reducing employment-related health inequities [11].

The macro-level framework indicates structural pathways through which health and health inequities associated with employment relations are produced and reproduced. The political power relations between the key political and economic actors are important upstream factors that determine the characteristics of labour market (e.g., labour regulation) and welfare state institutions and policies (e.g., social protection policies). In addition, the macro-level framework highlights the need for investigating the pathways through which the characteristics of the labour market and welfare state influence workers’ welfare and inequities in health according to employment relations.

The micro-level framework presents several micro-level pathways – specifically harmful working conditions and material deprivation – through which employment precariousness may be linked to poor health outcomes of workers, directly or indirectly. Harmful working conditions refer to occupational exposures, hazards, and risk factors such as physical, chemical, biological, ergonomic, and psychosocial hazards. Since these harmful working conditions and material deprivation are unequally distributed across employment relations, they may have an important effect on employment-related health inequities. The micro-level framework further describes several mechanisms at a more micro-level through which employment and working conditions and material deprivation affect health inequities, including increasing the risk of developing negative health behaviours (e.g., smoking and heavy alcohol use), psychosocial factors (e.g., job insecurity), physio-pathological changes (e.g., reduced height due to child labour, increased blood pressure and heart rate due to work-related stress), as well as through unequal access to health care services. Finally, cross-cutting issues like gender inequity, labour migration, and race discrimination are taken into consideration as integral dimensions of the macro- and micro-level frameworks.

## Methods

 We used a scoping review methodology and then conducted a modified e-Delphi survey to seek expert opinions to help validate our findings from the scoping review. The literature search focuses on empirical research addressing three main questions:

1. how does informal employment affect workers’ health?
2. how do the health and health equity implications of informal employment vary across countries and according to gender and other social factors such as social class, race/ethnicity, age, and migrant status?
3. what are policies or programmes to mitigate the adverse health and health equity implications of informal employment?

Using a scoping review methodology [12], we synthesized key themes in the existing literature, evaluated the types of evidence available in the field of public health, and identified emerging gaps in research. We used the PRISMA-ScR (PRISMA extension for Scoping Reviews) to guide us in the reporting of our scoping review findings.

### Data sources and searches

Scopus, Web of Science, and ProQuest databases were searched for peer-reviewed articles. Only peer-review literature was included; editorials, proposals, conference abstracts, magazine articles, and news articles were excluded. We also scanned references of a relevant article and grey literature that was retrieved from a grey literature database (Google Scholar) and targeted website searches of known organizations and institutions (e.g., ILO; The World Bank; WHO; Women in Informal Employment: Globalizing and Organizing (WIEGO)). The electronic search included articles published online between January 2015 and December 2019. The search strategy was limited to English language sources.

Given the scoping review objective of mapping out existing knowledge and identifying emerging gaps in research, the quality of research studies was not assessed. Accordingly, we included all the results meeting our inclusion criteria. The inclusion criteria for the articles included the following: (1) the article must be empirical research that examines the health or health equity implications of informal employment or that addresses specific policies or programmes to improve the health of informal workers; (2) the article must frame the problem as informal work. Articles addressing only precarious employment, a condition of employment that cuts across the formal and informal sectors, were excluded.

### Search strategy

Literature searches were based on title, abstract, and keywords. Two central themes of the research questions – informal workers and health – were combined to guide the search. Search terms within each theme were combined with the Boolean operator OR, and themes were combined using the Boolean operator AND. The search terms of informal workers were: “informal work*” OR “informal labour” OR “informal labor” OR “informal employment” OR “informal economy” OR “informal sector” OR “shadow economy.” The search terms of health were: “health” OR “well-being.” Two authors independently screened titles and abstracts and screened for full-text articles. Disagreement among the authors on the selection of literature was resolved through discussion until a consensus was reached among the authors.

### Literature synthesis

The synthesis included quantitative analysis (e.g., frequency analysis) of the empirical research in terms of country context and the types of study methods (i.e., qualitative, quantitative, or mixed methods) and qualitative analysis (e.g., thematic analysis) of the research purpose. As described above, the macro- and micro-level frameworks provide potential subjects of research to grasp how and why employment relations affect the health of workers and their families and how to intervene to reduce inequities in health according to employment relations and conditions. In our scoping review, the frameworks were applied deductively to existing literature to determine what is known and unknown about the links between informal employment and health and the potential mechanisms linking informal employment and health inequities. In other words, the micro- and macro-level frameworks established a priori were used as a conceptual guide to categorize literature into relevant components of the frameworks and identify gaps in research that require further empirical observations and testing.

### A modified e-Delphi survey

To validate our findings from the scoping review and receive feedback on additional research and policy gaps on the health and health equity implications of informal employment, we conducted a modified e-Delphi survey with experts including researchers, research funders, global institutions, and other users of such research. This is a group facilitation technique that seeks to obtain consensus of expert opinions on a specific topic through the use of structured questionnaires sent electronically to participants [13–15]. The authors determined the list of potential participants who could represent the diverse expertise and knowledge about informal work and its health, gender, and equity implications in HICs and LMICs based on a review of the grey and academic literature. An invitation to participate was sent via email to recruit potential participants. There were two rounds of surveys that were sent out on July 11, 2020 and January 12, 2021, respectively, where participants were asked to respond anonymously to the questions (see Additional file 1). Participants were requested to indicate the degree to which they agree or disagree with the research and policy gaps the authors identified based on a scoping review. They were requested to expand on their response, providing reasoning for their answer and/or additional information in a text box. Following the first round of responses, we synthesized the responses and returned the responses to the participants in summary form for review. Participants were allowed to change their responses (as needed) after reviewing those of other participants. The survey included participants who could represent the varying expertise and knowledge about informal work and its health, gender, equity implications in HICs and LMICs. The list of 64 potential participants was determined based on the scoping review of grey and academic literature and through targeted website searches of known organizations and institutions.

## Results

### Screening results

**Fig. 1:**
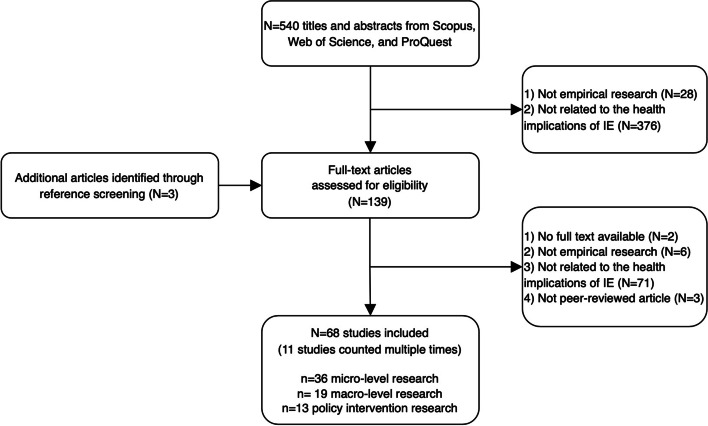
Flow diagram

The literature search resulted in 540 results (Fig. 1). After screening the abstracts for 540 potentially relevant papers, 28 were excluded for not being empirical research, 376 were excluded for not being related to the health implications of informal employment. After screening 139 potentially relevant full-text papers, 2 were excluded because they were not accessible in full text, 6 were excluded for not being empirical research, 3 were excluded for not being peer-reviewed, and 71 were excluded for not being related to the health implications of informal employment. A total of 57 studies were classified into one or more than one relevant category. Studies that have more than one relevant category were counted multiple times. Subsequently, 68 papers (with duplicates) were included for the scoping review.

### Characteristics of included studies

Of the 68 peer-reviewed articles, 9 were published in 2015 (13.2%), 11 in 2016 (16.2%), 14 in 2017 (20.6%), 13 in 2018 (19.1%), and 21 in 2019 (30.9%). Most studies were conducted in LMICs (51, 75.0%), including countries in Asia, Africa, and Latin America. Although only 17 were conducted in HICs including the United States, Canada, Chile, Greece, Spain, Japan, and countries in the European Union, the number increased from 4 to 2015 to 8 in 2019, indicating the growing research interest in informal employment and health in HICs as well as in LMICs. In terms of research methods, 41 (60.3%) were conducted using quantitative research methods, 23 (33.8%) using qualitative research methods, and 4 (7.4%) using mixed-methods.

### Scope and focus of included studies

Table 1 presents themes identified in our scoping review, which we have grouped into three broad categories, including micro-level, macro-level, and policy intervention research, based on the conceptual framework. Of the 68 peer-reviewed articles, 36 (52.9%) were papers that examined micro-level determinants of health in informal workers, 19 (27.9%) were papers that examined macro-level determinants of health inequities between workers in formal and informal employment, and 13 (19.1%) were research on policy interventions to mitigate the adverse health implications of informal employment. It should be noted that all empirical studies addressing cross-cutting issues such as gender inequity and labour migration were classified not only into relevant categories of micro-level research but also into macro-level research because they constitute structures of inequality embedded in our society. The gender category includes empirical studies that have analyse stratified by sex and/or examine the gender dimension of informal employment and its impact on health.

#### Micro-level research

Of the 36 micro-level research studies, the majority were studies conducted in LMICs (29, 80.6%). Diverse types of informal workers were studied in LMICs, including waste workers [16–21], trash pickers [22], clay artisans [23], taxi drivers [24], artisanal and small-scale gold miners [25], informal commerce workers [26], female beer promoters [27], domestic workers [28], informal fisheries [29], and informal workers in the construction and manufacturing industry [30–37]. The remaining micro-level studies were conducted in LMICs [38–43] and HIC contexts [9], rather than focusing on a specific type of informal workers, and analyzed survey data or injury reports to examine the difference in health outcomes between workers in informal and formal employment.

The majority of the 36 micro-level research studies (24, 66.7%) explored occupational exposures, hazards, and risk factors as an important mechanism linking informal employment to poor health outcomes. Of the 24 studies, most explored the poor working conditions present in the workplaces, such as exposure to physical, chemical, biological, and ergonomic hazards [17–19, 21, 22, 28, 31, 33–37, 44, 45]. There were 3 studies that focused on psychosocial risk factors, such as lack of control and high demands, and their association with health [26, 32, 39]. Some studies investigated the lack of knowledge and awareness of occupational safety and health risks on the part of workers and employers [16, 17, 20, 30] and poor safety practices such as the non-completion of safety training and non-use of personal protective equipment at work [17, 20, 25, 29, 36]. These are actions at the micro level that can help identify and mitigate occupational exposures, hazards, and risk factors in the workplaces.

Of the 36 micro-level research studies, 7 explored barriers to accessing health care [22, 27, 42–46], 1 quantitative study explored differential health effects of informal employment by income groups [47], and 1 quantitative study explored behavioural risk factors such as obesity, smoking, and alcohol drinking leading to poor health outcomes in taxi drivers in Thailand, most of whom were engaged in informal employment [24].

#### Macro-level research

Of the 19 macro-level research studies, 14 explored the intersection between gender and informal employment, including 7 and 7 studies in LMIC [28, 29, 43, 48–51] and HIC contexts [44, 45, 47, 52–55], respectively. It is notable that, among a total of 10 studies published in HIC contexts (without duplicates), 6 studies focus on the health and well-being of informally employed domestic workers [44], informal caregivers [47, 52–54], and recent immigrant women in informal employment [45]. This reflects the different realities of informal employment in LMICs and HICs: while in LMICs informal employment is a dominant source of employment, in HICs informal employment is rather concentrated in marginalized groups of society such as women, immigrants, and migrant workers. Also, the recent trend of emphasizing the gendered nature of informal employment in both LMICs and HICs indicates that paid/unpaid domestic and care work in the informal economy that has been perceived as being the responsibility of women (i.e., their family obligations) and undervalued socially and economically is becoming increasingly recognized.

Of the remaining 5 studies of macro-level research, 2 investigated the intersection between labour migration and informal employment in Greece [46] and United States [45]. 1 study analyzed how employment protection legislation moderates the effect of temporary and informal employment on the subjective well-being of the workers across European countries [56]. The remaining 2 studies conducted political analysis of the reform of the universal health coverage in Ethiopia [57] and of expanding healthcare access and financial protection to people in poverty and informal sector workers in Mexico and Turkey [58]. The latter two political analyses are categorized as macro-level research as they focus on examining the power relations between key political and economic actors and their implications on the health care reforms.

#### Policy intervention research

Of the 13 policy intervention research studies, all were conducted in LMICs with the purpose of either evaluating a specific policy intervention or exploring the experiences of workers or key informants with existing policies or programmes. Of these, 9 were in relation to extending health insurance to informal workers [59–67], 3 were about extending occupational health and safety services to informal workers [68–70], and 1 was about the implementation of the right to refuse dangerous work in South African mines [71].

**Table 1:** Summary of themes

Category	Theme	Pathway	N. of paper
Health implication	Micro determinants of work-related health inequity (*N* = 36)	Harmful working conditions - occupational exposures, hazards, and risk factors (physical, chemical, biological, ergonomic, and psychosocial hazards)	24^a^
		Material deprivation & economic inequalities	1^a^
		Access to health care (Health system)	6^a^
		Behavioral, psychosocial, and physio-pathological pathways	1
		No specific mechanism	3
	Macro determinants of work-related health inequity (*N* = 19)	Political power relations; intermediary labour market and welfare state policies leading to health inequities	3
		Gender	14^a^
		Labour migration	2^a^
Policy intervention (*N* = 13)	Health insurance policy		11
	Occupational health and safety		3
	Right to refuse dangerous work		1

^a^Papers were counted multiple times into relevant categories of research

### Health and health equity implications of informal employment

Findings from our scoping review are mixed on the health and health equity implications of informal employment. Informal employment does not necessarily lead to poorer health outcomes than formal employment. Our scoping review suggests that more research is needed to further understand the various mechanisms through which informal employment affects health in different countries and for different groups of informal workers.

#### Negative health implications of informal employment

The negative impact of informal employment on the subjective well-being of individuals is empirically corroborated by studies in Colombia [40], Mexico [38], and Central American countries [51]. Most notably, Lopez-Ruiz, Artazcoz [51] used a multidimensional measurement of informal employment, including absence of an employment contract, lack of social security coverage, or employment status, and found that not having social security coverage is the strongest predictor of poor subjective health and mental health for informal workers in both women and men. The adverse health impacts of informal employment are also supported by the aforementioned micro-level research studies in LMICs that describe occupational exposures, hazards, and risk factors, lacking knowledge and awareness of occupational hazards, and poor safety practices in specific types of informal work [16–22, 25, 26, 30–37, 39].

Additionally, the lack of social security coverage prevented the informal workers from seeking care at a medical institution. Direct and indirect costs, such as income foregone and the time to visit a medical doctor, were cited as major barriers to accessing health care. It was common across these studies that informal workers were heavily reliant upon informal ways of dealing with health problems, rather than through formal access to hospitals, including being dependent on medicines they purchased from the drugstores alone [27], self-medication [22], and herbal medicine [42]. A study in a HIC setting illustrates how migrant workers in the informal sector were frequently subject to invisible prejudice and discrimination at a public hospital and thus preferred private healthcare and to seek medical opinions from relatives or informal networks [46].

#### Taking gender and labour market and welfare state institutions into consideration 

Some studies provide empirical evidence that is counter to the negative health implications of informal employment. For example, in the 27 European Union countries, Julià, Belvis [9] found no significant difference in health outcomes between informal employees and permanent and temporary employees in formal employment, despite the informal employees having significantly worse working conditions and higher employment precariousness than the formal employees. The authors provide a potential explanation that the study results are likely to be driven by the healthy worker bias, i.e., those who survive in the informal economy despite poor working conditions are likely to be healthier.

Other studies attempt to account for the differences in the potential health implications of informal employment by groups of workers and welfare states. Most notably, employment informality has differential gendered effects. For example, in countries like South Africa where women experience the “double-burden” of income earning and family care, the increasing formality in employment brings greater health benefits to women than to men [50]. However, this protective effect of employment formality in women does not seem to hold in a study conducted in Chile [55]. In Chile, informal employment was significantly associated with poor subjective health and mental health for men but not for women. The authors provide a potential explanation that the working conditions for women in formal employment fall short of the standards of decent work because of the influence of neoliberal policies and thus are insufficient to have a protective effect on health. In the same vein, it is plausible that in LMICs where nation-states may have limited capacity to enforce occupational safety and health regulations, workers in formal employment may have health and safety problems as often as those in informal employment.

Furthermore, the adverse health effects of informal employment can differ by welfare state regimes. Rodriguez-Loureiro, Vives [48] examine how welfare state regimes influence the relationship between informal employment and health in the six Spanish-speaking countries in the Central American region. The results show that, in highly ‘familialist’ countries, characterized by limited state provision of welfare, weak social policies and high reliance on families for the provision of care, women in formal employment do not have a significantly better health outcome than those in informal employment. The authors suggest that this is potentially because women in highly ‘familialist’ countries experience a similar burden of care and domestic work regardless of their employment relations and share basic employment precariousness. In the same vein, Lopez-Ruiz, Benavides [49] highlight that health inequity between women in formal and informal employment can be reduced through the provision of both labour market and welfare state policies that can ensure decent work (e.g., full-time employment) and reduce the burden of unpaid care responsibilities at home.

Lastly, a case study explored the experiences of individuals providing unpaid, home-based renal care in rural British Columbia, Canada [52]. A feminist political economy lens allowed the authors to contextualize the experiences of informal care providers within the domestication of health care as well as a shift towards smaller government through privatization. This study also highlights that the informalization of labour is a prominent feature of broad, neo-liberal policy trends that attempt to shift the substantial amount of work and responsibility from state institutions to individual workers.

### The politics of health care reform for informal workers

Few studies discussed the politics of expanding access to health care for informal workers at a more macro level. Lavers [57] analyzes the Ethiopian pathway to health insurance expansion for informal workers. By drawing on the ‘Adapted Political Settlements’ framework, the author highlights that the centralisation of power within the ruling coalition during 2001–12 was an important driver of the reform. The ruling party’s commitment to self-reliance and resource mobilisation for development aligned with the policy idea of health insurance, and the dominance of power enabled overcoming opposition against health insurance expansion. Harris [58] explored the politics of policy adoption to provide healthcare access and financial protection for people in the informal sector in Mexico and Turkey. The author highlights that conservative political parties played critical roles in adopting the reform policy while labor unions and left-wing parties opposed the reform. Both Lavers [57] and Harris [58] point out that the pathways of the health care reforms for informal workers in developing economies were different from that of advanced industrial countries with a large working class and a high level of formality in labour market. That is, unlike the advanced industrial countries, left-wing political parties and labor unions did not drive the social policy reforms in LMICs context. Macro-level empirical studies, despite rarely conducted, identify key political and economic actors and their interactions that are crucial to the development of upstream labour market and welfare state policies leading to health inequities. The political analysis provides a deeper understanding of not only the political economic causes of health inequities at a macro-level but also of the interventions to reduce these inequities in different political, economic, social contexts.

### Policy interventions to improve health of informal sector workers

In LMICs, the extension of health insurance to informal sector workers remains a key challenge. Different health insurance schemes and enrollment strategies were adopted by the governments to extend the coverage to informal sector workers and improve the retention of members, including the quasi-mandatory enrollment strategies in Tanzania [67] and mandatory micro health insurance in Nigeria [66]. While a study conducted in Bangladesh found that the healthcare utilization is significantly higher in the insured of a community-based health insurance scheme than the uninsured, in other studies enrollment rates only temporarily increased despite improved access to health care [66, 67]. The barriers to the enrollment and low retention rates among informal workers include, but not limited to, poor governance [64], unaffordable premium rates [63], and a lack of awareness and insurance literacy [59, 61].

Because informal workers remain excluded from the health and safety regulatory system, diverse interventions were attempted to extend occupational health and safety to informal workers, including the development of a health and safety program by non-governmental organizations with urban street vendors in South Africa [68] and by community stakeholders in rural Thai settings [69] and awareness program on various occupational health hazards among the ragpickers in India [70]. Even though informal workers are given the right to refuse dangerous work as in the case of South African mine workers [71], significant gaps in implementation were identified due to the predominant practice of non-confrontational consultation with supervisors.

### Expert opinions from a modified e-Delphi survey

We received 14 responses from 64 potential participants in the first round and 8 responses in the second round despite repeated follow-up. The low response rate is potentially due to the COVID-19 pandemic that affected the academic community personally and professionally. In general, participants indicated agreement with the four research gaps identified by the authors based on the scoping review: (1) a lack of consensus on the definition of informal employment within literature reviewed including health studies; (2) an underdeveloped level of understanding of different health and health equity implications of informal employment by countries, groups of workers, and sectors; (3) less attention is being given in research to explore the intersections between gender and informal employment; (4) dearth of policy studies on the relationship between informal work and health.

Most of the participants agreed that there is a lack of consensus on the definition of informal employment in health studies (64.3% and 87.5% in the first and second round, respectively). In particular, participants indicated that there is also considerable confusion amongst terms such as informal, precarious, and non-standard employment in research and policy contexts. Participants emphasized that these terms overlap but are not synonymous and thus need to be used based on a precise definition and not be used interchangeably. There was a high level of consensus on the usefulness of the concept of informal employment in highlighting health inequities across employment relations and working conditions (78.6% and 87.5% in the first and second round, respectively), the need for more comparative policy research (71.4% and 62.5%, respectively), more qualitative health research (92.9% and 87.5%, respectively), and research on the intersection between gender and informal employment (78.6% and 62.5%, respectively).

The lack of consensus among respondents was only in the area of informal care and domestic work. The majority in the first round and half of the participants in the second round (78.6% and 50%, respectively) agreed that unpaid care and domestic work need to be more meaningfully integrated into the concept of informal employment. Some participants commented on the need for distinguishing between paid domestic work and unpaid care work for one’s own family. While paid domestic work is already integrated into the concept of informal employment, unpaid care work remains a contentious issue. Some argued that reproductive work (unpaid care and domestic work) needs to be recognized as work or employment, because their ‘work’ enables others to work and remain productive and thus is crucial for the health of the economy and society. Others argued that unpaid care of one’s own family should not be integrated within the broad definition of informal employment. It was emphasized that policies that could improve the working conditions and level of social protections for the informal workforce are not of the same nature as policies that should be developed for unpaid caregivers for members of their own family.

## Discussion

This scoping review synthesized available academic and grey literature on the health and health equity implications of informal employment, which is of considerable importance to further understand the pre-existing challenges faced by informal workers and the unequal impacts of the COVID-19 pandemic globally. The modified e-Delphi survey showed overall consensus about research gaps we identified in literature. The majority of participants in the modified e-Delphi survey agreed that there is a lack of consensus on the definition of informal work and a need to improve conceptual clarity.

The application of well-established conceptual frameworks in the work literature strengthened our analysis of the health and health equity implications of informal employment. These micro- and macro-level frameworks held up well by providing a useful lens for characterizing and analyzing the structural pathways that can produce and reproduce associations between health or health equity and employment relations, as well as the complex pathways between employment relations and health, the mechanisms linking health and gender inequities and employment relations, and the multiple levels at which interventions can be targeted. The findings of this study showed evidence that, although all informal workers share some forms of fundamental vulnerability, including harmful working conditions and limited access to health and social protections, the related health implications vary according to the sub-groups of workers (e.g., gender) and the country context (e.g., type of welfare state or labour market characteristics). Given the different contexts in which labour informality is unfolding, we could also learn a great deal from context-sensitive studies – contexts including country, sociodemographic characteristics of informal workers, and labour market and welfare state institutions and policies. Moreover, the interactions between informal employment and various axes of social inequality such as, but not limited to, gender, social class, and immigration status, should be investigated in future studies [51].

In this review, we identified relatively few articles in HICs published between 2015 and 2019 that associated informal employment with health. On the one hand, this implies that there is a perceived low frequency of informal employment in HICs that deters the investigation of the health and health inequity implications of informal employment [8, 9]. On the other, it remains to be seen whether other concepts such as precarious employment or non-standard employment may be more useful or relevant to capturing the work experience of vulnerable workers in HICs. The rise of informal work arrangements in HICs requires a much more nuanced understanding of the informal employment by countries, groups of workers, and sectors.

However, despite the relative paucity of literature in HIC contexts, our findings suggest that there has been a growing interest in studying informal employment and its health implications in HICs as well as in LMICs. Most notably, most of the reviewed studies in HICs shed light on the gendered nature of informal employment with a focus on examining the health of informally employed domestic and care workers. This trend can be understood in the context of what some scholars have already suggested that ‘a new wave of informalization’ is emerging in many HICs where informal employment had seemingly disappeared in the process of modernization and remained invisible in official statistics due to its illegal nature [72]. Moreover, the increasing recognition of female immigrants or migrant workers from LMICs, who predominate in care and domestic work in the informal sector of HICs, highlights the interconnected nature of the informal world of work between HICs and LMICs. This also reminds us of the universal nature of the SDGs in that the interconnected goals apply to all countries rather than only intended for action in LMICs.

As previously noted, some participants in the modified e-Delphi survey raised a concern about the conceptualization of care and domestic work, especially in relation to incorporating unpaid care of one’s own family into the concept of informal work and resultant vagueness or conceptual fuzziness. Nevertheless, some of the included studies highlight the crucial issue of women’s unequal share of unpaid care and domestic work and its implications on women’s experiences in the labour market [48, 50, 55]. In the context of the “double-burden” of income earning and family care imposed predominantly on women, the increasing formality in employment brings greater health benefits to women than to men [50]. However, this protective effect of employment formality is generated only if the increasing formality in employment ensures better social protection that can address women’s unpaid care work [48, 55]. It seems valid to incorporate into the study of informal work unpaid care and domestic work for one’s own family, which is inextricably intertwined with women’ opportunities to engage in paid employment and the type and quality of jobs they can take.

The majority of existing research in LMICs focused on describing harmful working conditions, i.e., occupational exposures, hazards, and risk factors, such as physical, chemical, biological, ergonomic, and psychosocial hazards. While this type of research remains vital, less is known about the policies that are needed to protect informal workers and promote their health and well-being. We found few policy studies, and these were restricted to either occupational health and safety issues or the extension of health insurance to informal workers in LMICs. It is necessary to investigate the differential impacts of policy and program interventions, which are not only targeted to occupational health and safety and health insurance, but also employment, education and care policies, and the mechanisms that link interventions addressing informal employment to the health of informal workers. Given the gendered nature of informal employment, we also need more gender- and equity-focused analyses of policies that can shape the experience of informal workers.

This study combining the results from a scoping review and e-Delphi survey is not without limitations. Firstly, our search was limited to empirical studies published online between January 2015 and December 2019 and written in English. By limiting the date range and language of publication, we may have missed some relevant literature. Although we found more articles were published in LMICs than in HICs, it is still possible that we did not capture the diverse and multi-faceted problems in non-English-speaking countries. Future research is needed to extend the period of publication and expand the scope of the literature review and e-Delphi survey to other languages to thoroughly integrate the research findings and enhance comparability across countries. Secondly, we included “health” and “wellbeing” into the search terms and thus were unable to rule out the possibility of inadvertently excluding relevant empirical literature that do not necessarily have the terms in the title, abstract, or keywords. However, our attempt to use well-established theoretical frameworks to map out existing knowledge can meaningfully contribute to illuminating research gaps and potential directions of future research. Thirdly, we had a low participation rate for the modified e-Delphi survey. In addition, while we acknowledge that research on informal work can be influenced by several factors including researcher and institutional interests, we did not include these types of questions in the e-Delphi survey. Despite these limitations, our findings provide a solid ground for understanding both some forms of fundamental vulnerability shared by all informal workers and different health and health equity implications of informal employment by countries, groups of workers, and sectors in pre-pandemic period. As suggested by participants in the modified e-Delphi survey, future studies should examine which combination of social, economic and health policies work best to protect the health of informal workers, for whom, under what contextual circumstances, and with what effects on health and health equity during the pandemic and beyond.

## Conclusions

 This scoping review highlighted the growing production of research and policy studies focused on informal employment and their health and health equity impacts in HICs as well as in LMICs. The health and health equity implications of informal employment have been further amplified during the COVID-19 pandemic. Empirical studies conducted in many countries have shown that informal workers are exposed to harmful working conditions and poor access to health care. Without underestimating such harsh realities facing informal workers, our scoping review illuminates that the health and health equity implications of informal employment can be markedly different by groups of workers (e.g., gender, immigrant status) and by countries (e.g., labour market and welfare state characteristics). In this respect, further studies are required to improve our understanding of the various mechanisms through which informal employment affects health in different countries and for different groups of informal workers. In particular, global studies need to take an explicit gendered perspective, especially due to the gendered nature and health effects of informal employment. In making the transition from the informal to the formal economy, countries should consider the ways in which informal employment intersects with gender and other social factors such as social class, race/ethnicity, age, and migrant status.

## Supplementary Information

**Additional file 1.**

## Data Availability

All data analysed during this study are included in this published article.

## Abbreviations

HICs: High-income countries
LMICs: Low-and-middle-income countries
ILO: International Labour Organization
WHO: World Health Organization
UN: United Nation
SDG: Sustainable Development Goal
