# Supplementary material for: How does informal employment affect health and health equity? Emerging gaps in research from a scoping review and modified e-Delphi survey
Source: Int J Equity Health. 2022 Jun 21;21:87. doi: 10.1186/s12939-022-01684-7 (PMC9208971; doi:10.1186/s12939-022-01684-7)
Supplement: Supplementary file 1 — Additional file 1. [file 12939_2022_1684_MOESM1_ESM.pdf]

Health and Informal Work Globally: Research Priorities Survey Questions[i]

Context for Study

The future of work is a growing area for research and policy locally, regionally, nationally, and globally. New technologies like online labour platforms and Artificial Intelligence (AI), as well as the intensification of longstanding labour trends like employment precariousness are creating new challenges for workers, their health, and for health and social policy systems. The concepts of the informal sector, informal employment, and informal economy were first adopted in 1950s to illustrate economic development within the 'developing' world. Within the current context of technological progress and globalization, informal employment has increasingly become a global phenomenon. Informed by a synthesis of academic and grey literatures, a background paper was developed to explore the concept of informal employment in a historical context. The paper summarizes current trends in research and policy related to informal workers' health, identifies frameworks that conceptualize informal employment and the pathways linking informal employment to health, as well as emerging knowledge gaps related to the health, gender and health equity implications of informal employment.

Based on our review, we identified four emerging research gaps in the relationship between informal employment and health.

1. A lack of consensus on the definition of informal employment within literature reviewed including in health studies.
2. An underdeveloped level of understanding of different health and health equity implications of informal employment by countries, groups of workers, and sectors.
3. Less attention is being given in research to explore the intersections between gender and informal employment.
4. A dearth of policy studies on the relationship between informal work and health.

The paper informed the development of the following survey. The goal of this modified Delphi process is to validate our findings and receive feedback on additional research and policy gaps related to informal employment and its intersections with health.

Below, we have summarised the research gaps. Please review the background paper before completing the brief survey. We have also noted specific sections in paper that relate to particular statements.

By completing and submitting responses to this survey, I consent to participating in this research study.

a) Yes
b) No

Please select the response that most accurately describes your work.

a) I am a researcher
b) I am a research funder
c) I use the research
d) I work for a global institution

Please indicate the extent to which you agree or disagree with the following 9 statements. For each statement, there is a text box where we welcome you to expand on your response, providing reasoning for your answer and/or additional information.

The scale is as follows:
1 = Strongly disagree
2 = Somewhat disagree
3 = Uncertain (neither agree nor disagree)
4 = Somewhat agree
5 = Strongly agree

Emerging Research Gaps

A) There is a lack of consensus on the definition of informal employment within literature reviewed including in health studies.

Building on the ILO's leadership on operationalizing the concept of employment in the informal economy, the ILO parameters to classify informal employment (e.g., social security coverage) have become increasingly popular in health studies. However, a consensus of the definition of informal employment in health studies, distinct from the concepts of precarious employment or non-standard employment, is necessary to allow for the assessment of the magnitude and trends of the informality and its health and health inequity implications in a comparative perspective across countries and regions (for more information, please see pages 6-11 of the background paper).

1. There is a lack of consensus on the definition of informal employment.

Strongly disagree 1 2 3 4 5 Strongly agree

Comments:

2. Informal work as a distinct concept is useful for highlighting health inequities across employment relations and working conditions.

Strongly disagree 1 2 3 4 5 Strongly agree

Comments:

3. The definition of informal employment varies depending on the country or region in which the concept is being considered.

Strongly disagree 1 2 3 4 5 Strongly agree

Comments:

B) There is an underdeveloped level of understanding of different health and health equity implications of informal employment by countries, groups of workers, and sectors.

Although all informal workers share some forms of fundamental vulnerability, the related health implications vary according to the sub-groups of workers engaging in informal employment. The sub-groups of informal workers may have different levels and experiences of employment precariousness. The mechanisms that link informal employment to health may vary according to employment relation and working conditions. Further research is required to uncover the unique health-related vulnerabilities affecting different groups of workers in informal employment (for more information, please see page 16-17).

4. Sub-groups of informal workers include low-wage workers, migrants, undocumented workers, self-employed (i.e. those operating informal sector enterprises), unpaid family workers, and domestic workers. To what extent do you agree that these are the most common sub-groups of informal workers?

Strongly disagree 1 2 3 4 5 Strongly agree

Comments:

5. Based on different sub-groups you are familiar with, what are the **top three** health-related vulnerabilities affecting these different groups of workers engaging in informal employment? Please provide a brief rationale for each.

Comments:

The existing literature pays less attention to understanding high-income countries' context for informal work. Given the different contexts in which these labour trends are unfolding, we could learn a great deal from comparative (within and between country) policy research. This research could examine which combination of social, economic and health policies might work best, for whom, under what contextual circumstances, and with what effects on health equity (for more information, see pages 17-18 of the background paper).

6. More comparative policy research is needed to better understand the context for informal work with regard to the different social, economic and health related policies.

Strongly disagree 1 2 3 4 5 Strongly agree

Comments:

7. More qualitative research is needed to better understand the lived experiences of informal workers in different country contexts. Please elaborate on the type of qualitative research studies, as needed.

Strongly disagree 1 2 3 4 5 Strongly agree

Comments:

C) *Less attention is being given in research to explore the intersections between gender and informal employment.*

Female informal workers are often in more vulnerable forms of employment statuses such as contributing family workers and domestic workers. We need research which goes beyond simply noting the differences between men and women into probing the root causes of the gendered inequalities in the informal employment and their health implications (for more information, please see pages 14-15 and 18 of the background paper).

8. Unpaid care and domestic work need to be more meaningfully integrated into the concept of informal employment.

Strongly disagree 1 2 3 4 5 Strongly agree

Comments:

D) There is a dearth of policy studies on the relationship between informal work and health.

The majority of existing research on informal work and health has focused on describing the associations between informal employment and different health outcomes. While this type of research remains vital, less is known about the policies that are needed to protect informal workers and promote their health and well-being. It is also necessary to investigate not only policies which are targeted at the extension of either occupational health and safety or health insurance coverage to informal workers, but also employment, education and care policies. Given the gender inequalities in informal employment, we need more gender- and equity-focused analyses of policies that can shape the experience of informal workers (for more information, please see pages 18-19 of the background paper).

9. More research is needed on the health and gender impacts of employment, education and care policies on informal workers.

Strongly disagree 1 2 3 4 5 Strongly agree

Comments:

10. Are you aware of any countries that currently take health, gender and/or equity considerations under advisement when developing or evaluating policies that we could learn from? Please elaborate.

Comments:

11. Of the four gaps listed in the background paper *(lack of consensus on the definition of informal employment; an underdeveloped level of understanding of different health and health equity implications of informal employment by countries, groups of workers, and sectors; less attention given in research to exploring the intersections between gender and*

informal employment; and a dearth of policy studies on the relationship between informal work and health), which **two gaps** do you believe are the most important to address and why? Please elaborate.

12. Additional comments

Thank you for participating in our survey. Results will be summarized and shared back with all participants in a subsequent round.

---

[i] Note: The same questions were used in both rounds of the survey.
